# Supplementary material for: Evaluation of the Small Heat Shock Protein Family Members HSPB2 and HSPB3 in Bladder Cancer Prognosis and Progression
Source: Int J Mol Sci. 2023 Jan 30;24(3):2609. doi: 10.3390/ijms24032609 (PMC9917356; doi:10.3390/ijms24032609)
Supplement: Supplementary file 1 [file ijms-24-02609-s001.zip › Supplementary Table S1.pdf]

Supplemental Table S1. Cox regression analysis for MIBC (T2-T4) patients' overall survival (OS) following treatment.

	MIBC (T2-T4) overall survival (OS)				
	**Univariate analysis**				
**Covariant**	**HR[a]**	**95% CI[b]**	***p*-value[c]**	**Bootstrap BCa 95% CI[d]**	**Bootstrap *p*-value[c]**
HSPB2					
Low expression	1.00				
High expression	1.698	0.539-5.353	0.366	0.499-6.508	0.339
HSPB3					
Low expression	1.00				
High expression	1.131	0.365-3.507	0.832	0.313-3.955	0.831
**Tumor Stage**					
T2	1.00				
T3-T4	7.066	1.532-32.58	0.012	2.136-103.4	0.007
**Age** (Continuous variable)	1.005	0.954-1.058	0.849	0.956-1.063	0.811
	**Multivariate analysis for *HSPB2*[e]**				
**Covariant**	**HR[a]**	**95% CI[b]**	***p*-value[c]**	**Bootstrap BCa 95% CI[d]**	**Bootstrap *p*-value[c]**
HSPB2					
Low expression	1.00				
High expression	1.193	0.359-3.966	0.774	0.315-7.404	0.778
**Tumor Stage**					
T2	1.00				
T3-T4	8.744	1.728-44.26	0.009	$2.651\text{-}9.45 \times 10^5$	0.003
**Age** (Continuous variable)	1.032	0.958-1.112	0.406	0.948-1.164	0.493
	**Multivariate analysis for *HSPB3*[e]**				
HSPB3					
Low expression	1.00				
High expression	1.167	0.372-3.666	0.791	0.265-7.645	0.792
**Tumor Stage**					
T2	1.00				
T3-T4	9.076	1.804-45.65	0.007	$2.776\text{-}1.43 \times 10^6$	0.004

**Age** (Continuous variable)	1.031	0.957-1.110	0.423	0.934-1.188	0.508

[a] Hazard Ratio
[b] 95% confidence interval of the estimated HR
[c] Calculated by test for trend. Bootstrap *p*-value is based on 1000 bootstrap samples
[d] Bootstrap 95% confidence interval of the estimated HR based on 1000 bootstrap samples
[e] Multivariate analysis adjusted for tumor stage and patients' age
