# Supplementary figures and images for: Evaluation of the Small Heat Shock Protein Family Members HSPB2 and HSPB3 in Bladder Cancer Prognosis and Progression
Source: Int J Mol Sci. 2023 Jan 30;24(3):2609. doi: 10.3390/ijms24032609 (PMC9917356; doi:10.3390/ijms24032609)

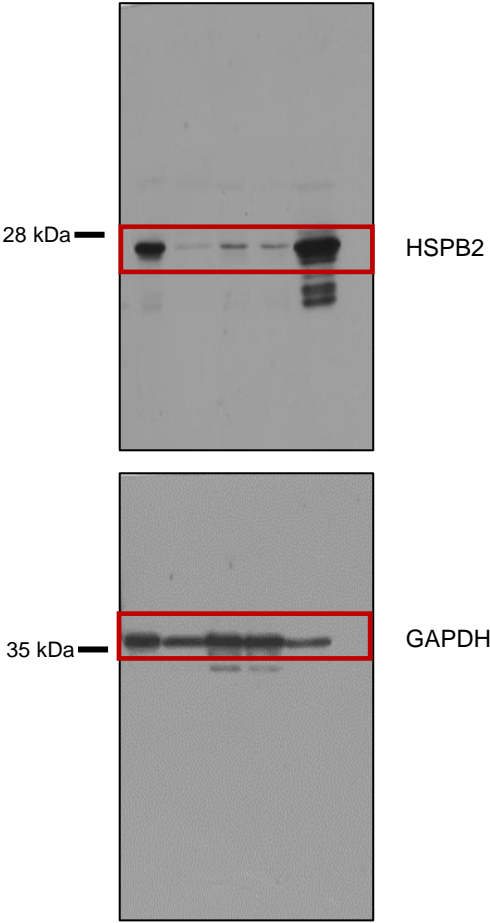

**Figure S1**

Supplement: Supplementary file 1 [file ijms-24-02609-s001.zip › Supplementary Figure S1.pdf]
